# Electroencephalography-Based Brain–Computer Interface System Using Tongue Movement Imagery for Wheelchair Control

**DOI:** 10.3390/s26072211

**Published:** 2026-04-02

**Authors:** Theerat Saichoo, Nannaphat Siribunyaphat, Bukhoree Sahoh, M. Arif Efendi, Yunyong Punsawad

**Affiliations:** 1 School of Informatics, Walailak University, Nakhon Si Thammarat 80160, Thailand; theerat.sa@wu.ac.th (T.S.); nannaphat.sir@wu.ac.th (N.S.); bukhoree.sa@wu.ac.th (B.S.); 2 Informatics Innovative Center of Excellence, Walailak University, Nakhon Si Thammarat 80160, Thailand; 3 Department of Nuclear Engineering and Engineering Physics, Faculty of Engineering, Universitas Gadjah Mada, Yogyakarta 55281, Indonesia; arif.e@ugm.ac.id

**Keywords:** brain–computer interface, brain-controlled wheelchair, electroencephalography, motor imagery, tongue movements, machine learning

## Abstract

Brain–computer interfaces (BCIs) are essential in assistive technologies to restore mobility in individuals with motor impairments. Although electroencephalography (EEG)-based brain-controlled wheelchairs have been extensively studied, most tongue-controlled systems rely on physical tongue movements, intraoral devices, or limited offline commands, which reduces the usability and comfort. This study introduces an EEG-based tongue motor imagery (MI) BCI for intuitive and entirely mental wheelchair control. By leveraging preserved motor function and the cortical representation of the tongue, the system enables natural four-directional control through imagined tongue movements. Six imagined tongue actions—touching the left and right mouth corners, the upper and lower lips, and producing left and right cheek bulges—were designed to elicit alpha-band event-related desynchronization (ERD) patterns over the tongue motor cortex. EEG data were collected from 15 healthy participants using a 14-channel consumer-grade EMOTIV EPOC X headset. Alpha-band ERD features were extracted and classified using linear discriminant analysis, support vector machine, naïve Bayes, and artificial neural networks (ANNs). Simpler command sets yielded the highest accuracy: two-class tasks achieved 76.19%, while the performance decreased with increasing task complexity. The ANN achieved superior results in multi-class scenarios. The proposed tongue MI method offers initial support for developing a BCI control strategy for assistive technology; however, further improvements in classification techniques, user training, and real-time validation are needed to improve the robustness and practical usability.

## 1. Introduction

Recent research on brain–computer interfaces (BCIs) has advanced steadily, with an increasing emphasis on practical applications in medicine [1,2] and in domains such as education [3], sports [4], and entertainment [5]. Among the available brain signal acquisition systems, electroencephalography (EEG) remains the most commonly used technique because of its reliability and established clinical applications. Furthermore, modern EEG devices are becoming more user-friendly and efficient for BCI research [1,6]. In addition, functional near-infrared spectroscopy has been developed [7,8] to promote wider adoption. Hence, advanced research laboratories continue to explore invasive and non-invasive BCI technologies to enhance the therapeutic effectiveness and expand the real-world applications of these technologies [9,10,11,12].

For BCI-based assistive technologies, researchers have developed tools and systems aimed at enhancing the quality of life and supporting both the physical and mental health of people with disabilities [13,14]. Advances in mobility-assistive technologies, which include powered wheelchairs and prosthetics, have been achieved using BCI technology [15,16]. This enables a direct connection between the brain and external devices [17,18], where users can control them autonomously [19,20].

EEG-based BCIs and event-related desynchronization (ERD) and synchronization (ERS) [21,22] are triggered by motor imagery (MI) tasks. Steady-state visually evoked potentials (SSVEPs) [23,24] are generated by repetitive visual stimulation. Both these paradigms are widely used in BCI research. SSVEP-based methods are highly effective and require little training; however, they depend on external stimuli and can be negatively affected by visual fatigue, which can impair performance [25,26]. In contrast, MI involves the natural motor-related thought processes of the user, causing task-related EEG changes that appear as gradual increases or decreases in sensorimotor rhythms [27,28]. Owing to this natural control approach without external stimuli, MI-based BCIs are popular because of their versatility and have demonstrated success in assistive and rehabilitation applications [29,30]. MI-based BCIs involving limb movements have proven highly efficient during training sessions and depend on advanced classification algorithms. In addition to limb-related imagery [31,32], researchers have explored the challenge of distinguishing between MI tasks involving multiple body parts. Tongue movement imagery has gained attention because the tongue has a disproportionately large cortical representation in the sensorimotor cortex and often remains under voluntary control even in individuals with severe motor impairments. Consequently, tongue MI has been studied as a promising approach, and research has assessed its feasibility and potential use in BCI systems [33,34].

Previous studies have investigated the connection between tongue movements and EEG signals to support the development of EEG-based BCI systems. Görür et al. measured the glossokinetic potential (GKP) using EEG and extracted delta and theta waves using a discrete wavelet transform (DWT). They combined these with the mean absolute value (MAV) and power spectral density (PSD) features by employing decision trees (DTs) and k-nearest neighbors (kNN) for classification, with the kNN + MAV method yielding the best performance [35]. Kæseler et al. used the common spatial pattern (CSP) to distinguish movement from stillness, classifying three frequency bands with linear discriminant analysis (LDA) and achieving 71% accuracy for the two classes through 10-fold cross-validation and receiver operating characteristic analysis [36]. Subsequently, they extended this to four-command classification by removing the EMG/GKP noise and applying LDA, support vector machine (SVM), random forest (RF), and multilayer perceptron (MLP), realizing an accuracy of 75.6%, as confirmed by ANOVA [37]. Gulyás and Jochum-sen studied ear-EEG for detecting hand and tongue movements using SVM, k-NN, RF, and LDA and achieved 83% accuracy for tongue control because of its proximity to the ear. However, this was conducted offline, and there is a need for online applications [38]. Based on the EEG changes observed during tongue movement, further research is required to develop MI for tongue-MI-based BCI systems. Giannopulu and Mizutani examined brain signals during the imagined movements of the hands, feet, and tongue and hypothesized that tongue imagery is linked to communication. Network connectivity and correlation analyses revealed notable parieto-frontal activation during tongue imagery, unlike hand and foot imagery [33]. La Touche et al. found that combining action observation (AO), visual mirror feedback (VMF), and facial exercises reduced the pressure sensitivity and enhanced the mental imagery, which supports their potential in orofacial rehabilitation for dysphagia [39]. Dos Santos et al. proposed a signal-processing technique for MI-BCI using hand, foot, and tongue imagery, thereby applying modulation filtering and regularized CSP to extract features, followed by a two-stage classification with LDA and naïve Bayes (NB). The tongue MI achieved the highest accuracy of 77.16% [40]. Gong et al. analyzed brain connectivity during MI to predict BCI usability using four feature extraction methods along with SVM and WPLI for network analysis. Tongue imagery demonstrates strong bilateral connectivity in the frontal and midbrain regions [41].

Although research on tongue MI-based BCIs is increasing, most studies remain confined to movement-based paradigms, offline analyses, or low-dimensional command sets (e.g., binary or four-class), limiting their practical assistive value. Tongue function is often preserved in individuals with severe motor impairments and is closely linked to communication, offering advantages over limb-based MI. Developing a six-command non-invasive tongue-MI system therefore addresses the need for higher control complexity and advances the practicality of assistive BCI applications.

Recent studies on EEG-based tongue movement and MI BCIs (Table 1) highlight the tongue as a promising modality for assistive control due to its preserved function and strong cortical representation. However, many systems still rely on actual movements, intraoral devices, or limited offline commands, which restrict their practical use. In response, a fully non-invasive multi-command tongue-MI system is needed. Recent advances in deep learning, such as CNNs combined with wavelet-based EEG analysis, have shown high accuracy in classifying tongue-related motor tasks [42], offering a promising direction for enhancing MI decoding and enabling more intuitive BCI control.

This study investigates a six-command tongue MI-based brain–computer interface (BCI) for multi-directional wheelchair navigation. Different cortical activation patterns associated with tongue-imagery tasks are decoded by extracting the EEG features related to tongue motor activity, enabling hands-free control for assistive applications. The main contributions of this work are summarized as follows:

- Development of a six-command tongue MI paradigm for hands-free multi-directional wheelchair control.
- Identification and decoding of distinct cortical activation patterns associated with various tongue-imagery tasks using EEG signals from a low-cost EEG system.
- Extraction and analysis of tongue-related motor features to classify commands.
- Offline evaluation demonstrating the feasibility and reliability of tongue MI as an alternative control method.
- Provision of evidence supporting the potential integration of tongue MI-based BCI into future real-time assistive navigation systems.

The remainder of this article is organized as follows: Section 2 introduces a new tongue movement imagery paradigm and examines the EEG activity recorded during these tasks. Section 3 describes the methods used, including the system architecture, EEG signal collection and processing, feature extraction, and classification techniques. Section 4 reports the experimental results, focusing on the performance and effectiveness of the offline testing. Section 5 and Section 6 present the conclusions, findings, and possible future directions for the proposed system.

## 2. Materials and Methods

**Figure 1:**
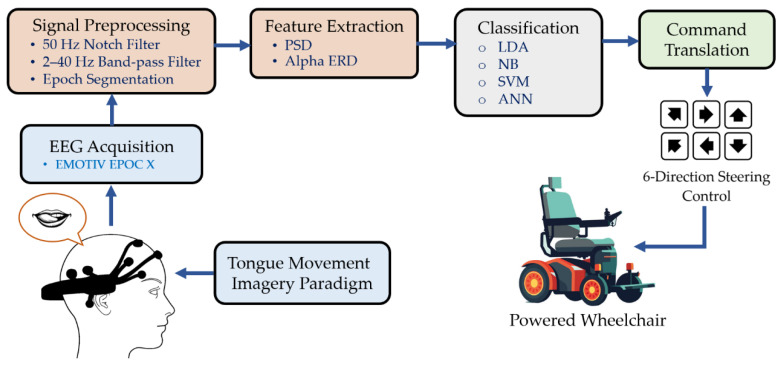
Flowchart of proposed brain-controlled wheelchair using tongue movement.

Conventional BCI systems based on MI comprise a movement imagery paradigm; EEG signal collection; signal processing algorithms that include preprocessing, feature extraction, and classification; translation of decoded signals into control commands; and applications such as wheelchair operation, robotic arms, electrical appliances, communication aids, and rehabilitation (Figure 1).

In this study, we developed a method that uses tongue movement imagery to build a BCI system focused on tongue motion. This approach is particularly useful for patients with severe paralysis who retain voluntary tongue control and sensory perception. It enables the development of assistive devices such as communication interfaces, environmental control systems, and neurorehabilitation tools. This system can expand command options and enhance the accessibility and functionality of BCI technologies for individuals with motor impairments by utilizing tongue MI.

MI involves mentally rehearsing movements without actual execution, generating consistent neural signals utilized in BCI research. During movement planning or visualization, motor areas exhibit oscillatory patterns called ERD/ERS, which reflect changes in neural activity. Imagining a movement on one side produces ERD/ERS in the opposite (contralateral) sensorimotor cortex, such as C3 and C4, in the 10–20 system, with the initial ERD followed by ERS in the 10–12 and 18–26 Hz frequency bands [43]. Tongue movement control stems from the facial region of the primary motor cortex, with tactile and proprioceptive inputs from the tongue transmitted to the primary somatosensory cortex in the parietal lobe.

### 2.1. Proposed Tongue Movement Imagery Paradigm

We propose a tongue MI paradigm for an EEG-based BCI system to generate control commands for wheelchair navigation. Tongue-based MI tasks are advantageous for individuals with severe motor impairments because the tongue retains fine motor control even in cases of spinal cord injury or paralysis. Tongue movements produce distinguishable EEG patterns that can be effectively decoded for assistive-control applications.

The proposed tongue MI paradigm enables four-directional wheelchair control through purely mental tasks, which offers a noninvasive, natural, and fatigue-resistant approach. The tongue motor cortex is located in a well-defined region of the sensorimotor area of the brain, and EEG systems such as the EMOTIV EPOC X can effectively capture reliable ERD and ERS patterns during imagined tongue movements, offering more consistent responses compared with conventional limb-based MI.

**Figure 2:**
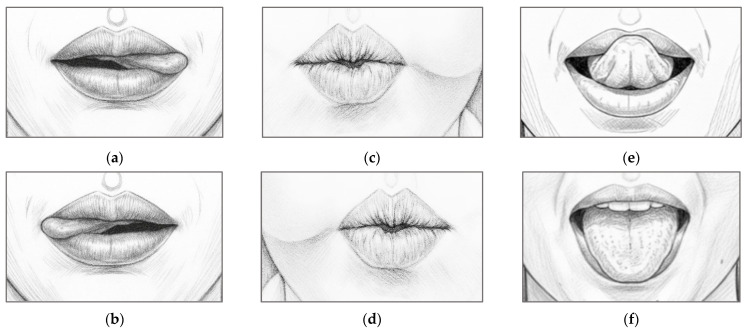
Tongue movement imagery paradigms: (**a**) tongue tip touching the left corner of the mouth; (**b**) tongue tip touching the right corner of the mouth; (**c**) tongue tip pressing against the left cheek bulge; (**d**) tongue tip pressing against the right cheek bulge; (**e**) tongue tip touching the upper lip; and (**f**) tongue tip touching the lower lip.

To promote intuitive and efficient control, the paradigm defines six distinct tongue MI tasks, each corresponding to a specific directional command: left-45°, left-90°, right-45°, right-90°, forward, and backward. As summarized in Table 2, these tasks are mapped to symbolic commands and associated with wheelchair control. The six MI tasks were organized into three movement patterns based on the spatial nature of the imagined tongue gesture to facilitate user training:

- Pattern 1 involves lateral tongue movements where participants imagine touching the corners of the mouth with the tongue tip (Figure 2a,b, corresponding to left-45° and right-45° turns).
- Pattern 2 also involves lateral gestures but requires imagining the tongue pressing against the inner cheek bulge (Figure 2c,d, corresponding to sharper left-90° and right-90° turns).
- Pattern 3 includes vertical tongue movements involving the imagination of touching the upper and lower lips with the tongue tip (Figure 2e,f, mapped to forward and backward commands, respectively).

This design aims to enable users to control the wheelchair accurately and responsively with minimal training, using spatially consistent and easily visualized gestures. The mapping between the imagined tongue movements and directional commands leverages natural spatial associations, enhancing the intuitiveness and reducing the cognitive workload. The turning angles are modulated by the degree of imagined lateral movement (e.g., corner of the mouth vs. cheek bulge), while vertical gestures control forward and backward motion (Table 2).

### 2.2. Experimental Task

**Figure 3:**
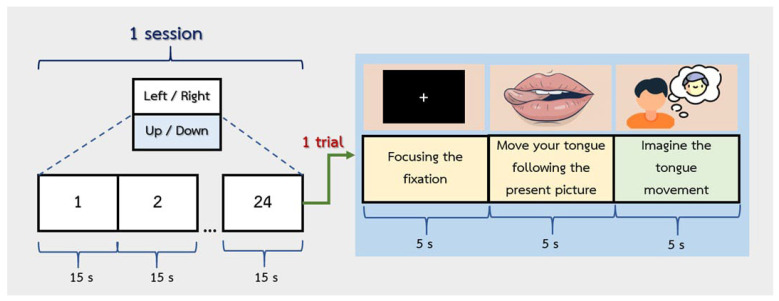
Task experiment for the motor imagery via tongue movement paradigms.

The participants performed three distinct tongue movement patterns. Each pattern included two sessions, and the experimental flow is shown in Figure 3. Each session comprised 24 trials. At the beginning of each trial, participants were instructed to fixate on a cross (+) without moving for 5 s, which served as the baseline period. This was followed by a 5 s execution phase, during which participants physically moved their tongue according to the direction displayed on the screen. Subsequently, a 5 s MI phase was conducted, in which participants imagined performing the same tongue movement without any actual motion. This trial structure enabled comparison between the baseline, execution, and imagery conditions for subsequent analysis. A 5 min rest was provided between the two sessions for each pattern to minimize fatigue, and a 10 min break was provided before moving on to the next pattern. The three patterns (left, right, up, down, forward, and backward (Figure 2)) were presented in a randomized order for each participant. Each participant completed 144 trials (24 trials × 2 directions × 3 patterns).

### 2.3. EEG Acquisition and Processing

**Figure 4:**
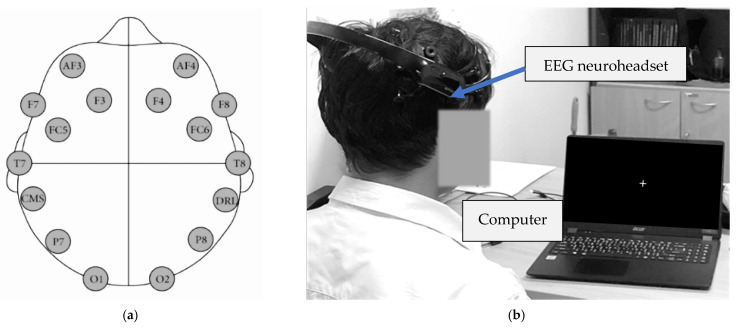
(**a**) Electrode placement locations based on the international 10–20 system. (**b**) Example scenario during the experiment showing a participant wearing the EMOTIV EPOC X neuroheadset while fixating on a visual cue.

EEG signals were acquired using a 14-channel EMOTIV EPOC X neuroheadset (EMOTIV Inc., San Francisco, CA, USA; accessed 25 September 2025), a flexible and low-cost device selected to evaluate the feasibility of scalable brain–computer interface (BCI) systems for assistive applications and translational research. Electrode placement followed the international 10–20 system and covered key regions linked to motor processing, including AF3, AF4, F3, F4, F7, F8, FC5, FC6, T7, T8, P7, P8, O1, and O2. Electrodes FC5, FC6, T7, and T8 are known to detect motor-related EEG activity and are often used in MI analysis with similar EEG headsets [44]. Electrodes M1 and M2 served as reference points (Figure 4a). The EEG signals were recorded at a sampling rate of 256 Hz.

During preprocessing, the EEG signals were first cleaned with a 50 Hz notch filter to remove the power-line interference, followed by a 2–40 Hz bandpass FIR digital filter to reduce motion artifacts while preserving the mu (8–12 Hz) and beta (13–30 Hz) rhythms associated with MI, consistent with established EEG preprocessing practices [45]. After filtering, EEG channels over the motor cortex were selected for further analysis. Subsequently, the signals were segmented into a 5 s baseline period, during which participants maintained visual fixation without movement, and a 5 s MI period triggered by visual cues (Figure 4b). A frequency-domain analysis was performed to calculate the PSD to evaluate sensorimotor rhythm modulation during MI tasks. Based on the observed spectral characteristics, relevant features, such as the mu and beta band powers, were extracted to capture the neural activity changes between the baseline and imagery conditions. All filtering, channel selection, segmentation, spectral analysis, and feature extraction procedures were performed in MATLAB (MathWorks Inc., Natick, MA, USA; R2021a) using the EEGLAB toolbox (version 2023) [46].

A total of 15 healthy subjects (nine females and six males; aged 20–24 years) participated in the study. None of the participants had any known neurological conditions, and they provided informed consent before participation. Confidentiality related to personal information was maintained throughout the study period. Ethical approval was granted by the Human Research Ethics Committee of Walailak University (Project No. WU-EC-IN-1-217-66, approval no. WUEC-23-250-01, 5 October 2023), and all procedures adhered to the Declaration of Helsinki, as well as CIOMS and WHO ethical guidelines.

### 2.4. Observations of EEG with Different Tasks

The tongue motor area is located in a well-defined region of the primary motor cortex, specifically along the lateral side of the precentral gyrus. Imagined tongue movements performed without actual muscle activation lead to characteristic neural changes in this region. The ERD and ERS reflect shifts in oscillatory activity during MI. Furthermore, we focused on the 10–12 Hz range, which corresponds to the dominant mu rhythm associated with motor and MI processes and enhances the sensitivity to tongue motor activation by minimizing interference from general visual alpha signals.

**Figure 5:**
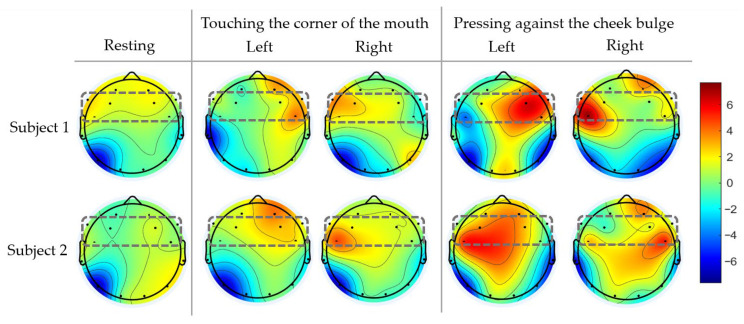
Examples of topographic brain maps of alpha-band ERD (10–12 Hz) for Subjects 1 and 2 during tongue-related MI tasks, including imagined left and right tongue movements that involve touching the corner of the mouth or pressing against the cheek bulge. The dashed box indicates the region of interest (ROI) corresponding to the sensorimotor cortical area.

**Figure 6:**
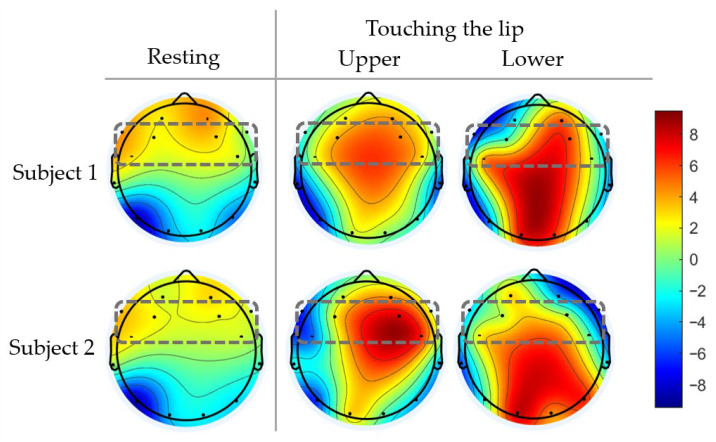
Examples of topographic brain maps of alpha-band ERD (10–12 Hz) obtained from EEG recordings of Subjects 1 and 2 while they imagined touching the tongue tip to the upper or lower lip. The dashed box indicates the region of interest (ROI) corresponding to the sensorimotor cortical area.

Segmented EEG signals were visualized using topographic brain maps to observe the cortical activation patterns associated with tongue MI. The grand-average topographic distribution of absolute alpha-band power (10–12 Hz) across all trials was computed for each participant to assess the neural responses during the tongue MI tasks. Figure 5 and Figure 6 show that distinct feature patterns were concentrated in the frontal–central cortical regions, which confirms that these areas were key contributors to the detection of tongue MI.

Figure 5 presents the topographic maps of the alpha-band ERD (10–12 Hz) for subjects 1 and 2 during four tongue MI tasks: imagining movements toward the left and right corner of the mouth and cheek bulge. The ERD was computed as the percentage power change from the baseline and averaged across trials for each condition. Contralateral activation is evident, with a stronger ERD at FC5 during right-side imagery and at FC6 during left-side imagery. The cheek bulge imagery produced stronger a ERD than the mouth corner imagery, suggesting stronger neural engagement. Additional desynchronization at F3/F4 and AF3/AF4 indicates the involvement of frontal and prefrontal regions in motor planning and internal simulation.

Figure 6 shows the topographic distributions of the alpha-band ERD (10–12 Hz) for subjects 1 and 2 during the imagery of touching the upper and lower lips. Similar to Figure 5, the percentage power change from the baseline and the average across trials for each condition are presented. Both imagery tasks elicited ERD over the frontal–central regions; however, the lower-lip imagery resulted in stronger and more spatially widespread desynchronization. The hemispheric activation patterns varied with the imagery direction and between subjects, with subject 1 exhibiting more lateralized activation and subject 2 showing a more bilateral distribution. These differences suggest that lower-lip imagery engages broader cortical networks, likely due to the increased imagined movement amplitude or muscular involvement.

Together, Figure 5 and Figure 6 demonstrate the task-dependent modulation of spatial activation across the AF3/AF4, F3/F4, and FC5/FC6 electrodes, as well as variations in hemisphere symmetry and asymmetry. Tongue MI tasks involving higher imagined movement or effort, such as cheek-press and lower-lip imagery, consistently produced stronger and more spatially distinct alpha-band ERD patterns compared with mouth-corner and upper-lip imagery. These trial-averaged spatial features provide a more reliable basis for decoding multiple commands in real-time BCI applications. To exploit these characteristics effectively, ERD-based features can be combined with spatial filtering and fast robust classifiers. Moreover, because ERD patterns vary across users and tasks, online adaptation or incremental learning is essential for maintaining long-term reliability. Incorporating spatial filtering efficient classification and adaptive updating can support consistent six-command tongue MI control for practical wheelchair navigation, while subject-specific cortical patterns may further enhance personalized BCI performance.

## 3. Proposed Algorithms

### 3.1. Feature Extraction

Based on preliminary studies [47], both alpha- and beta-band ERD patterns were observed during tongue MI, with beta-band results summarized in Table A1. However, alpha-band ERD was selected as the primary feature due to its clearer spatial discriminability and more consistent performance (Table 3 and Table A2), particularly in simpler tasks. Although beta showed selective improvements in some multi-class conditions, no significant overall differences were found, and alpha achieved slightly higher accuracy in key two-command tasks, supporting its use as a reliable baseline.

Observations of the mu rhythm (Section 2) showed clear alpha activity at AF3/AF4 and significant alpha ERD at FC5/FC6 during lateralized tongue tasks, confirming contralateral activation (Figure 5 and Figure 6). The asymmetry at F3/F4 indicates involvement in motor planning. Due to the limited number of electrodes in the EEG device, the effective use of advanced spatial filtering methods, such as common spatial patterns (CSPs), is limited. Additionally, previous work [37] demonstrated that PSD features over short time windows enable efficient feature extraction.

As illustrated in Figure 2, the EEG data were segmented into 5 s resting and MI periods for each trial to detect the tongue movement imagery. To extract features in the frequency domain, the PSD was estimated using the Welch periodogram method. Each 5 s segment was divided into 1-s windows with 50% overlap, and 256-point FFT was applied to each Hamming window segment. The resulting PSDs were averaged across windows to obtain a stable frequency representation. In this pilot study, alpha-band power (10–12 Hz) was used as a discriminative feature as this band is associated with sensorimotor rhythm desynchronization during MI tasks [48]. The ERD was calculated as the percentage change in power between the resting and MI periods:

(1) $${ERD}_{i}=\frac{P_{Bl,i}-P_{Im,i}}{P_{Bl,i}}\times 100,$$

where $P_{Bl,i}$ and $P_{Im,i}$ represent the average alpha-band power (10–12 Hz) of channel *i* during the baseline and MI phases, respectively. The following channels were included in the analysis: AF3, AF4, F3, F4, F7, F8, FC5, FC6, T7, T8, P7, P8, O1, and O2. The final feature vector for each trial was constructed by concatenating the ERD values from these channels in a fixed order, supporting compact and lightweight classification for online BCI processing [41].

Since 14 EEG channels were included in the analysis, each trial produced one ERD feature per channel, resulting in a 14-dimensional feature vector (1 × 14) per trial. Therefore, for a dataset containing N trials, the input feature matrix to the classifier was structured as N × 14, where each row represents one trial, and each column corresponds to the alpha-ERD feature of a specific channel. This low-dimensional representation reduces the computational complexity and supports real-time BCI implementation.

### 3.2. Classification Methods

After preprocessing and feature extraction, feature vectors were used for two-, four-, and six-class classification tasks, each representing a different tongue MI command. Classification was conducted using the MATLAB Classification Learner App, which provides standardized workflows for training and validating machine learning models. The classification pipeline was designed to provide a transparent reproducible baseline evaluation by using participant-level data partitioning, a simple holdout validation strategy, and default model hyperparameters. All samples were grouped according to each individual participant to prevent data leakage between the training and testing sets. A participant-level split was applied, with data from each individual assigned to either the training or testing set. The dataset was divided using a 70/30 holdout scheme, with about 70% of participants in the training set and the remaining 30% reserved for independent testing. This approach ensured that the performance metrics reflected the model’s ability to generalize to unseen individuals rather than learn subject-specific patterns.

Within the training set, the model evaluation employed the default 10-fold cross-validation in the Classification Learner App to assess the stability and minimize the performance variability. Cross-validation was performed solely on the training data, while the independent test set was reserved for final evaluation, without nested cross-validation or additional resampling. All classifiers used the default hyperparameters to maintain consistency and fairness, focusing on the discriminative ability of the extracted features and channels rather than on performance optimization.

Traditional machine-learning methods were chosen for their interpretability and suitability for small EEG datasets, while deep learning requires more data and resources. This approach balances the performance, data limits, and interpretability for effective tongue MI classification [49]. Four common classification algorithms used in tongue-based BCI systems [35,36,37,38] were employed:

- Linear discriminant analysis (LDA): A linear classifier that constructs decision boundaries by modeling the distribution of each class and maximizing the class separability.
- Naïve Bayes (NB): A probabilistic model based on Bayes’ theorem that assumes feature independence, offering fast computation and reasonable performance for biomedical data.
- Support vector machine (SVM): A margin-based classifier that constructs an optimal separating hyperplane using linear kernels by default in our setting.
- Artificial neural network (ANN): A shallow feedforward network with one hidden layer of 10 neurons, trained using scaled conjugate gradient backpropagation with Nguyen–Widrow weight initialization, a maximum of 1000 epochs, and early stopping after six consecutive validation failures (minimum gradient = 1 × 10^−7^).

The classification performance was evaluated using the accuracy, reported as the mean and standard deviation across the 10-fold cross-validation within the training set, while the independent holdout test set was used for the final performance assessment, following the established EEG-based classification evaluation protocols [50]. This preliminary trial-level analysis aimed to establish baseline results using physiologically interpretable features and low-complexity classifiers suitable for real-time BCI applications.

## 4. Experimental Results

For each tongue MI pattern and channel configuration, the dataset was organized with consistent trial labels corresponding to six directional imagery tasks. Each participant completed 24 trials per command, resulting in 360 samples per command across 15 participants; this yielded a total of 2160 samples, with 360 samples per class in the six-class configuration, ensuring a class-balanced dataset.

The data were evaluated under three classification schemes:

(1) Two-class system: Paired directional imagery tasks were grouped into binary comparisons: LL–LR, CL–CR, and LU–LD. Each comparison included two commands (360 samples per class), totaling 720 samples per pattern.
(2) Four-class system: Two configurations were evaluated: LL–LR–LU–LD and CL–CR–LU–LD. Each configuration contained four commands with 360 samples per class (1440 samples total), maintaining class balance.
(3) Six-class system: All tongue imagery tasks (LL, CL, LR, CR, LU, LD) were classified as separate commands, totaling 2160 samples, with 360 samples per class in a balanced distribution.

All datasets were classified using the MATLAB Classification Learner App (Section 3.2). For the ANN classifier, the network weights were randomly initialized and optimized with scaled conjugate gradient backpropagation. A consistent labeling strategy and standardized classification pipeline allowed for the systematic evaluation of feature sets, channel configurations, and classifier performance under subject-independent testing conditions.

### 4.1. Multi-Class Tongue Motor Imagery Classification Using Alpha ERD

In the command classification analysis, we focused on the alpha frequency band and assessed four machine learning algorithms: LDA, SVM, ANN, and NB. We evaluated three classification types of two, four, and six classes, as summarized in Table 3.

Three command patterns were defined for the two classes. Patterns 1, 2, and 3 include commands 1 and 2 (LL-LR), commands 3 and 4 (CL-CR), and commands 5 and 6 (LU-LD), respectively. In Pattern 1, LDA, SVM, and ANN achieved the highest accuracy of 76.19%, while NB achieved a lower accuracy of 61.90%. For Pattern 2, the ANN yielded the best accuracy (76.19%), followed by SVM, NB (71.43%), and LDA (66.67%). In Pattern 3, SVM achieved the highest accuracy of 76.19%, while LDA, NB, and ANN obtained 71.43%.

For the two-class classification, we defined three patterns: Pattern 1 included 45° left-turn and 45° right-turn commands (commands 1 and 2); Pattern 2 included 90° left-turn and 90° right-turn commands (commands 3 and 4); and Pattern 3 included moving forward and moving backward commands (commands 5 and 6). In Pattern 1, LDA, SVM, and ANN achieved the same average accuracy of 76.19%, whereas NB yielded a lower accuracy of 61.90%. For Pattern 2, the ANN achieved the highest accuracy of 76.19%, followed by SVM and NB at 71.43%, with LDA performing the lowest at 66.67%. In Pattern 3, SVM achieved the highest accuracy of 76.19%, while the remaining algorithms achieved 71.43% accuracy.

For the four-class classification, which included commands 1, 2, 5, and 6 (LL-LR-LU-LD), the ANN achieved the highest accuracy (67.44%), followed by LDA (58.14%), SVM (55.81%), and NB (48.84%). In the second pattern, which included commands 3, 4, 5, and 6 (CL-CR-LU-LD), the ANN performed the best (65.12%), with LDA, SVM, and NB reaching 60.47%, 58.14%, and 55.81%, respectively.

Finally, for the six-class classification, both the ANN and LDA achieved the highest average accuracy of 54.69%, followed closely by SVM at 53.12%, and NB achieved the lowest at 42.18%.

Additionally, the results for each command in Table 3 were analyzed based on each classification scheme. In the two-class system, LL and LR achieved the highest and most consistent accuracies with LDA, SVM, and ANN (approximately 75.0–77.6%), while NB performed worse (60.6–63.3%). For CL–CR, CR achieved higher accuracy than CL. LU consistently outperformed LD in the LU–LD comparison. In the four-class configurations, LL, LR, and LU yielded the same range of accuracies with the ANN, whereas LD consistently exhibited the lowest performance across all classifiers. A similar pattern was observed in the CL–CR–LU–LD configuration. In the six-class classification, LL and LU only achieved 56–59% accuracy across LDA, SVM, and ANN, while LR, CL, and CR showed moderate performance (51–56%). LD had the lowest accuracy (around 35–48%) across all classifiers. Overall, horizontal (LL and LR) and upward (LU) tongue movements were more distinguishable than downward (LD) movements.

To provide statistical validation of the observed performance differences, we evaluated the statistical significance of the command generation using LL–LR and CL–CR commands. A paired t-test showed no significant difference between LL–LR and CL–CR in the two-class classification (n = 60, *p* > 0.05). Furthermore, additional paired comparisons between the two-command schemes showed no significant differences between LL–LR and LU–LD (n = 60, *p* > 0.05) or between CL–CR and LU–LD (n = 60, *p* > 0.05). Similarly, no significant difference was observed in the four-class classification when comparing LL–LR–LU–LD with CL–CR–LU–LD (n = 60, *p* > 0.05). These results indicate that the choice between LL–LR and CL–CR does not significantly affect the classification performance. However, a significant difference was found between the two-class and four-class classification schemes (n = 120, *p* < 0.001), indicating that the number of command classes substantially impacted the system performance. The accuracy was higher in the two-class condition than in the four-class condition, reflecting the increased complexity of multi-class EEG signal classification. Table 3 also shows that the classification accuracy decreases as the number of commands increases.

To further investigate the differences between command schemes, pairwise statistical comparisons were conducted using paired t-tests. Effect sizes (Cohen’s d) and 95% confidence intervals were also calculated to measure the magnitude of the differences observed. Table 4 shows that most pairwise comparisons did not reveal statistically significant differences (*p* > 0.05), and the effect sizes were small (|d| < 0.3). For example, the comparison between LL–LR and CL–CR showed a mean difference of 1.19 with a small effect size (d = 0.083), while LL–LR and LU–LD exhibited virtually no difference (d = 0.000). Similarly, the comparison between the four-command schemes (LL–LR–LU–LD vs. CL–CR–LU–LD) yielded a small effect size (d = −0.25) and was not statistically significant (*p* = 0.061). These findings suggest that the differences in classification performance across command configurations are relatively minor and should be viewed as trends rather than definitive performance advantages.

### 4.2. Performance Evaluation of Alpha ERD-Based Classifiers for Tongue Motor Imagery

**Figure 7:**
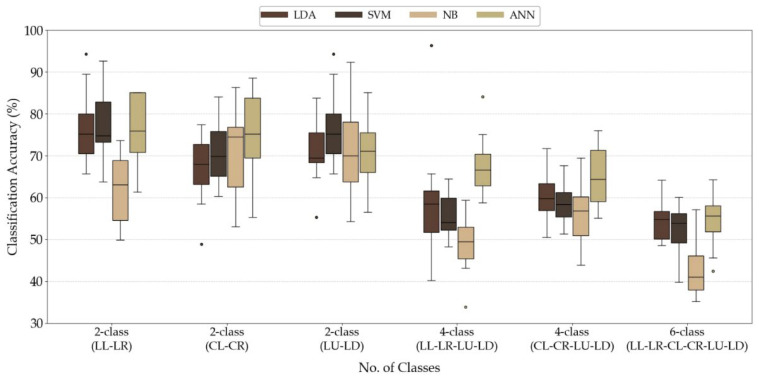
Results of the proposed classification methods across different class configurations. Boxplots show classification accuracy distributions, where the central line represents the median, boxes indicate the interquartile range, and dots denote outliers.

Figure 7 shows the classification accuracies for the six tongue MI patterns using LDA, SVM, NB, and ANN. The boxplots confirm that the two-class tasks achieved higher and more consistent accuracies than the four- and six-class tasks, reflecting the increased classification difficulty as the number of command categories increases. Across all patterns, the ANN achieved the highest mean accuracy in the two-class tasks, reaching approximately 76%, demonstrating the effectiveness of nonlinear models in capturing the complexity of tongue MI. In contrast, NB showed the lowest and most variable performance, particularly in the six-class condition.

Lateral turning commands (LL–LR and CL–CR) generally showed slightly more stable performance compared to vertical movements (LU–LD), although the differences were modest in the two-class configuration. The accuracy decreased across all algorithms when the commands were combined into four- or six-class tasks. SVM and NB experienced the most significant performance reductions, while the ANN maintained a relative performance advantage. In the four-class tasks, the ANN outperformed NB by approximately 10–20% and exceeded SVM and LDA by 7–12%. In the six-class scenario, the highest median accuracies (~54–55% for the ANN and LDA) remained below the commonly accepted threshold for reliable multi-command BCI control.

These results demonstrate that alpha-ERD-based classification is feasible for limited command sets, particularly for binary directional tasks. However, additional feature enhancement or hybrid classification strategies may be necessary to achieve higher reliability in six-class tongue MI control.

Table 5 shows the information transfer rate (ITR) based on the alpha-band ERD features for three command schemes using the proposed classifiers. The highest ITR values were seen in the two-class setup, where SVM and ANN achieved the best results (around 6.9 bits/min), with SVM reaching 7.36 bits/min for LU–LD. Lateral command pairs (LL–LR and CL–CR) also showed consistent performance across classifiers. When the number of commands increased to four and six, the ITR decreased across all models, indicating increased classification difficulty. The ANN generally maintained the best performance in multi-class schemes (about 4.38–5.82 bits/min), while NB showed the largest decrease and the lowest ITR values. Overall, these results suggest that alpha-band ERD features support effective information transfer for binary tongue MI control, but the performance declines as the number of commands increases.

## 5. Discussion

Based on Table 3, ANN and SVM performed best in the two-class tasks, with ANN achieving the highest accuracy for CL–CR and SVM for LU–LD, while NB consistently showed lower accuracy. The strong performance of LDA, SVM, and ANN suggests that alpha-ERD features contain partially separable information, particularly under binary conditions. Accuracy declined for all models as the number of classes increased to four, with ANN showing only a slight advantage. The combination of CL–CR with LU–LD yielded better performance than LL–LR with LU–LD, likely due to stronger alpha ERD responses. In the six-class task, accuracy decreased below 60% for all models, and differences between ANN and LDA became negligible, indicating that increased model complexity cannot compensate for the limited discriminative power of alpha-band ERD features. Moreover, the relatively large standard deviations across subjects reveal significant inter-subject variability in EEG responses, which may affect classification performance depending on the specific training and testing sets. This indicates that the results could be sensitive to subject selection and might not fully generalize to unseen individuals. Although leave-one-subject-out (LOSO) validation was not used in this study due to computational limits and dataset constraints, it is a more suitable evaluation method for EEG classification and will be considered in future work to ensure robust cross-subject generalization.

Figure 7 shows how the classification accuracy varied between the 15 participants. The accuracy declined with an increase in the number of command classes, with two-class tasks maintaining a higher accuracy, especially for LDA, SVM, and ANN. NB consistently performed worse and showed more variability, highlighting its limitations. The ANN still showed a somewhat better accuracy when the number of classes increased to four and six. However, the overall decline in performance across all algorithms suggests that relying only on alpha-band features may not be sufficient to identify more commands in tongue-MI-based BCI. Using only alpha-ERD features limits this study because it does not fully capture the neural dynamics of tongue MI, especially in complex tasks such as six-command control. This selection reduced the feature dimensionality, computational load, and latency, which makes the system suitable for real-time embedded-based BCI. Adding other frequency bands or advanced preprocessing can improve the accuracy; however, these methods often increase the processing cost and are not practical for low-power hardware platforms.

Furthermore, the information transfer rate (ITR) followed the same trend as accuracy, with the two-class configuration achieving the highest bitrates. Although more commands increase theoretical ITR, decreased accuracy in four- and six-class tasks lowered actual performance. ANN maintained a relatively higher ITR in multi-class settings, while NB showed the largest decline. These findings suggest that alpha-band ERD is effective for binary control, but improved feature extraction or hybrid approaches are necessary for dependable multi-command communication.

A comparison between alpha- and beta-band ERD features (Table 3 and Table A1) shows that alpha slightly outperformed beta in the 2-command LL–LR task, while results varied for CL–CR and LU–LD; meanwhile, beta showed selective improvements in 4-command schemes, particularly with SVM and NB. In the 6-command scheme, performance was nearly identical, and accuracy decreased as the number of commands increased, indicating that task complexity is the primary factor. Statistical analysis (Table A2) confirmed no significant differences (*p* > 0.05), although moderate-to-large effect sizes with wide confidence intervals suggest high variability. Overall, alpha performs slightly better in simpler tasks, while beta offers selective benefits in moderately complex scenarios; however, task complexity outweighs band choice, suggesting that single-band approaches may be insufficient and that combining alpha and beta features with advanced classifiers could improve robustness.

Compared to previous tongue-based BCI studies, the proposed tongue MI approach demonstrates initial feasibility with lower system complexity. For example, Kæseler et al. [36,37] reported higher accuracies with real-time tongue movement data, a larger number of EEG channels, and more advanced classification methods, achieving accuracies of 71–87.7% in two-class tasks and 43–62.6% in four-class tasks. Similarly, Gulyás and Jochumsen [38] achieved up to 82.5% accuracy by combining tongue movements with additional modalities. In contrast, the present study utilized a limited number of EEG channels from a consumer-grade headset, focused on alpha-band ERD features, and employed motor imagery rather than actual tongue movements. To further contextualize these differences, Table 1 presents a structured comparison of prior studies and the proposed method in terms of EEG channel configuration, feature extraction methods, task design, and evaluation protocols. These methodological variations are likely to contribute to the observed performance gap as each factor can significantly influence classification accuracy. In general, studies employing higher-density EEG systems, richer spatial–spectral features, and motor execution paradigms tend to achieve superior performance. Nevertheless, the simplified setup used in this study improves portability and usability, albeit at the cost of reduced accuracy, particularly in multi-class scenarios.

This study performed an offline assessment to evaluate the proposed features and classifiers, including their ITR. While these offline results provide valuable insights, they demonstrate limited effectiveness for real-time use. However, consistent patterns across subjects and task conditions indicate that decoding tongue motor imagery with lightweight alpha-band features and simple classifiers is possible. Further research is needed to explore factors that influence real-time performance, such as user adaptation, feedback variability, and system latency. Understanding these elements is essential for practical applications and could greatly impact overall system performance. The main limitations and practical considerations are summarized as follows:

1. The system was evaluated under offline conditions. Although real-time dynamics were not modeled, the observed performance trends provide meaningful insight into system behavior and feasibility.
2. Stable EEG headset placement and low electrode impedance are critical for maintaining consistent signal quality and classification performance.
3. User training and variability: Users require training to produce consistent tongue MI patterns. Inter-subject variability and potential fatigue effects indicate the need for adaptive calibration and performance monitoring.
4. Classification accuracy decreases as the number of commands increases, mainly because of overlapping feature representations rather than classifier limitations. Although performance with multiple commands is still lower than artifact-based BCI systems, tongue motor imagery offers more stable and consistent control than traditional limb MI, especially in binary tasks.
5. Hybrid control strategy: A hybrid approach is recommended to improve robustness, where tongue-based commands handle critical actions (e.g., stopping, turning) while additional decoding methods support higher-level navigation [51]. This balances accuracy and computational efficiency for real-time embedded applications.
6. The study included only healthy young adults, limiting direct generalization to individuals with motor impairments. However, prior studies indicate that tongue motor function is relatively preserved in such populations, supporting the potential applicability of tongue-based control strategies [52,53].
7. The use of alpha-band features alone is insufficient for reliable multi-class decoding as the achieved accuracies for four- and six-class tasks remain below the commonly accepted threshold for practical BCI applications [54]. This highlights the need for improved feature representations or complementary strategies to enhance performance while maintaining real-time feasibility.

## 6. Conclusions

This study presented a tongue MI-based BCI for wheelchair control that uses alpha-band event-related desynchronization (ERD) features from a consumer-grade EEG headset. The results demonstrate that tongue MI signals can be reliably decoded to produce basic control commands, especially in binary classification tasks, highlighting the potential of tongue MI as an alternative control method for assistive BCI systems. Although the accuracy decreases as the number of classes increases, the findings provide valuable insights into the feasibility and challenges of multi-class tongue MI decoding. In addition, the current evaluation was performed offline in a controlled setting and involved only healthy young adults; therefore, the results may not fully reflect real-world closed-loop performance or directly apply to individuals with motor impairments. This research suggests the possible application of tongue-based MI-controlled wheelchair using accessible EEG technology. Future work will focus on enhancing feature extraction and classification, as well as developing real-time closed-loop testing.

## Figures and Tables

**Table 1:** Research studies on EEG-based tongue movement and motor imagery BCIs.

Author	Paradigm	Method	Results	Contribution
Giannopulu and Mizutani [33]	MI: four-class MI with visual cues	Graph theory, LORETA, and coherence	Tongue MI shows strong connectivity changes (frontal/parietal networks)	Ear-EEG is a low-cost method for tongue/hand detection.
Görür et al. [35]	Voluntary tongue movement: touching left/right cheek walls for 6 s	Features: MAV and PSD Classifiers: DT and kNN	Maximum classification accuracy: 96.77% with kNN + MAV (frontal EEG)	Demonstrated a practical EEG-based tongue–machine interface.
Kæseler et al. [36]	Real tongue movement in four visually cued directions	LDA with 10-fold CV	Four-class: 43%, three-class: 55%, and two-class: 71%	Tongue-based BCI for assistive directional control.
Kæseler et al. [37]	Multi-direction tongue movements	LDA, SVM, RF, and MLP	Four-class: 62.6%, three-class: 75.6%, and two-class: 87.7%	Stimulus-independent pre-movement tongue BCI.
Gulyás and Jochumsen [38]	Tongue–palate movement + wrist extension	RF, SVM, kNN, and LDA	SVM: 82.5%, RF: 78.75%, LDA: 76.25%, and kNN: 67.5%	Ear-EEG is a low-cost solution for tongue/hand movement detection.
La Touche et al. [39]	Orofacial exercises with MI, AO, and VMF	RCT + repeated-measures ANOVA	AO + MI reduces pain sensitivity. AO shows the greatest increase in tongue strength	Demonstrates neural distinctiveness and MI feasibility for tongue BCIs.
dos Santos et al. [40]	MI of tongue, hands, and feet using visual arrows	Modulation filtering, CSP + Tikhonov, and LDA + Naïve Bayes	Classification accuracies between 77.16% and 90.27%	Validates MI/AO approaches in orofacial rehabilitation (TMD and dysphagia).
Gong et al. [41]	MI of tongue, left/right hand, and feet	PSD, DWT + WPD + CSP, and FBCSP + SVMICA preprocessing WPLI + graph theory	Performance strongly correlated with brain network metrics	Tongue MI as a predictor of MI-BCI usability based on brain connectivity.

**Table 2:** Proposed action for tongue movement imagery tasks.

Commands	Symbols	Actions	Output Commands
1	LL	Imagine the tongue tip touching the left corner of the mouth	Turn left 45°
2	LR	Imagine the tongue tip touching the right corner of the mouth	Turn right 45°
3	CL	Imagine the tongue tip pressing against the left cheek bulge	Turn left 90°
4	CR	Imagine the tongue tip pressing against the right cheek bulge	Turn right 90°
5	LU	Imagine the tongue tip touching the upper lip	Move forward
6	LD	Imagine the tongue tip touching the lower lip	Move backward

**Table 3:** Average classification accuracy across three command schemes using four classification models based on alpha-band ERD features (mean ± SD).

Scheme	Commands	Command	Average Classification Accuracy (%) (Mean ± SD)			
			Classification Model			
			LDA	SVM	NB	ANN
2	LL-LR	LL	77.2 ± 6.76	77.4 ± 7.06	63.3 ± 7.35	77.6 ± 9.27
		LR	75.1 ± 8.07	75.0 ± 6.77	60.6 ± 6.48	74.7 ± 6.63
		AVG	76.2 ± 7.42	76.2 ± 8.34	61.9 ± 6.92	76.2 ± 7.94
	CL-CR	CL	65.8 ± 5.47	70.6 ± 8.59	70.0 ± 8.16	75.1 ± 10.6
		CR	67.5 ± 8.17	72.3 ± 7.22	72.8 ± 10.8	77.2 ± 9.35
		AVG	66.7 ± 6.84	71.4 ± 7.91	71.4 ± 9.50	76.2 ± 9.96
	LU-LD	LU	74.5 ± 8.19	78.4 ± 7.30	73.9 ± 9.83	74.4 ± 6.15
		LD	68.2 ± 7.66	73.9 ± 8.32	68.9 ± 12.3	68.4 ± 9.35
		AVG	71.4 ± 7.93	76.2 ± 7.81	71.4 ± 11.1	71.4 ± 7.75
4	LL-LR-LU-LD	LL	62.2 ± 10.4	59.4 ± 4.45	53.9 ± 5.74	70.6 ± 5.78
		LR	60.0 ± 12.5	58.3 ± 4.88	50.0 ± 5.25	69.7 ± 6.10
		LU	59.2 ± 12.5	57.4 ± 5.19	49.7 ± 5.33	68.8 ± 5.22
		LD	51.1 ± 15.2	48.1 ± 6.56	41.5 ± 7.91	60.6 ± 9.54
		AVG	58.1 ± 12.6	55.8 ± 5.27	48.8 ± 6.06	67.4 ± 6.66
	CL-CR-LU-LD	CL	60.8 ± 4.61	58.7 ± 3.72	56.7 ± 4.27	66.7 ± 5.08
		CR	62.6 ± 5.44	60.0 ± 4.01	58.9 ± 6.23	66.7 ± 6.41
		LU	63.3 ± 6.24	61.6 ± 4.87	57.8 ± 6.89	67.2 ± 6.03
		LD	55.3 ± 8.23	52.5 ± 7.05	49.7 ± 10.8	60.2 ± 9.78
		AVG	60. 5 ± 6.13	58.2 ± 4.91	55.8 ± 7.05	65.2 ± 6.83
6	LL-LR-CL-CR-LU-LD	LL	59.2 ± 3.78	56.9 ± 4.38	46.1 ± 6.94	58.6 ± 6.13
		LR	56.7 ± 4.67	55.9 ± 6.02	43.6 ± 5.89	56.4 ± 4.51
		CL	53.1 ± 3.56	51.6 ± 4.34	41.7 ± 4.67	53.8 ± 5.05
		CR	55.3 ± 3.17	54.2 ± 4.85	42.2 ± 4.41	55.9 ± 5.07
		LU	56.1 ± 4.35	54.8 ± 5.28	44.1 ± 5.12	55.8 ± 4.35
		LD	47.8 ± 7.74	45.8 ± 8.66	35.3 ± 8.65	47.8 ± 8.74
		AVG	54.7 ± 4.55	53.2 ± 5.59	42.2 ± 5.95	54.7 ± 5.64

**Table 4:** Statistical comparisons between command schemes with effect sizes and 95% confidence intervals.

Comparison	Mean Diff (%)	95% CI	t(59)	*p*-Value	Cohen’s d
LL-LR vs. CL-CR	1.19	[−2.51, 4.88]	0.64	0.523	0.083
LL-LR vs. LU-LD	0.00	[−3.29, 3.29]	0.00	0.999	0.000
CL-CR vs. LU-LD	−1.19	[−4.97, 2.60]	−0.63	0.533	−0.081
LL-LR-LU-LD vs. CL-CR-LU-LD	−2.32	[−4.76, 0.11]	−1.91	0.061	−0.25

**Table 5:** Average information transfer rate (ITR) for different command schemes and classification models.

Scheme	Commands	Classifier	ITR (Bits/min)
2	LL-LR	LDA/SVM/ANN	6.92
	CL-CR	ANN	6.92
	LU-LD	SVM	6.92
4	LL-LR-LU-LD	ANN	5.82
	CL-CR-LU-LD	ANN	4.38
6	LL-LR-CL-CR-LU-LD	LDA/ANN	4.21

## Data Availability

The data presented in this study are available upon request from the corresponding author.

## Appendix A

**Table A1:** Average classification accuracy across three command schemes using four classification models based on beta-band ERD features (mean ± SD).

Scheme	Commands	Average Classification Accuracy (%) (Mean ± SD)			
		Classification Model			
		LDA	SVM	NB	ANN
2	LL-LR	72.7 ± 8.30	71.4 ± 7.14	71.0 ± 10.92	72.2 ± 6.46
	CL-CR	61.9 ± 9.11	71.4 ± 7.91	70.4 ± 6.24	66.7 ± 6.88
	LU-LD	71.4 ± 9.96	73.2 ± 6.84	61.9 ± 10.40	72.2 ± 9.91
4	LL-LR-LU-LD	61.9 ± 6.49	65.1 ± 5.88	58.1 ± 8.51	66.4 ± 7.01
	CL-CR-LU-LD	58.2 ± 7.57	61.9 ± 8.75	58.1 ± 6.89	61.9 ± 6.46
6	LL-LR-CL-CR-LU-LD	54.7 ± 8.70	52.8 ± 5.64	43.8 ± 5.06	54.2 ± 5.13

**Table A2:** Statistical comparisons between command schemes with effect sizes and 95% confidence intervals between alpha and beta band.

Command Schemes	Mean Diff (%)	95% CI	t(59)	*p*-Value	Cohen’s d
LL-LR	−4.75	[−21.18, 11.68]	−0.92	0.425	0.425
LU-LD	3.58	[−3.67, 10.82]	1.57	0.214	0.785
CL-CR	1.18	[−8.36, 10.71]	0.39	0.721	0.196
LL-LR-LU-LD	−6.98	[−14.37, 0.42]	−3.00	0.058	−1.50
CL-CR-LU-LD	−5.17	[−13.41, 3.06]	−2.00	0.139	−1.00
LL–LR–CL–CR–LU–LD	−1.92	[−4.99, 1.14]	−2.00	0.140	−0.999
